# Prevalence of GMC performance assessments in the United Kingdom: a retrospective cohort analysis by country of medical qualification

**DOI:** 10.1186/s12909-017-0903-6

**Published:** 2017-04-04

**Authors:** L. Mehdizadeh, H. W. W. Potts, A. Sturrock, J. Dacre

**Affiliations:** 1 University College London Medical School, Royal Free Hospital, room GF/664, Hampstead, London, NW3 2PF UK; 2 UCL Institute of Health Informatics, 222 Euston Road, London, NW1 2DA UK; 3 Royal College of Physicians, 11 St Andrews Place, Regent’s Park, London, NW1 4LE UK

**Keywords:** Primary medical qualification, General Medical Council, Performance assessment, Cohort study, Brexit

## Abstract

**Background:**

The demographics of doctors working in the UK are changing. The United Kingdom (UK) has voted to leave the European Union (EU) and there is heightened political discourse around the world about the impact of migration on healthcare services. Previous work suggests that foreign trained doctors perform worse than UK graduates in postgraduate medical examinations. We analysed the prevalence by country of primary medical qualification of doctors who were required to take an assessment by the General Medical Council (GMC) because of performance concerns.

**Methods:**

This was a retrospective cohort analysis of data routinely collected by the GMC. We compared doctors who had a GMC performance assessment between 1996 and 2013 with the medical register in the same period. The outcome measures were numbers experiencing performance assessments by country or region of medical qualification.

**Results:**

The rate of performance assessment varied significantly by place of medical qualification and by year; *χ* ^2^(17) = 188, *p* < 0.0001, pseudo-R^2^ = 15%. Doctors who trained outside of the UK, including those trained in the European Economic Area (EEA), were more likely to have a performance assessment than UK trained doctors, with the exception of South African trained doctors.

**Conclusions:**

The rate of performance assessment varies significantly by place of medical qualification. This is the first study to explore the risk of performance assessment by individual places of medical qualification. While concern has largely focused on the competence of non-EEA, International Medical Graduates, we discuss implications for how to ensure European trained doctors are fit to practise before their medical licence in the UK is granted. Further research is needed to investigate whether these country effects hold true when controlling for factors like doctors’ sex, age, length of time working in the UK, and English language skills. This will allow evidence-based decisions to be made around the regulatory environment the UK should adopt once it leaves the EU. Patients should be reassured that the vast majority of all doctors working in the UK are competent.

## Background

The demographics of United Kingdom (UK) doctors has been changing in recent years and may change further following a referendum decision to leave the European Union (EU) [1]. We are in a period of heightened political discourse about immigration and supranational trade agreements across the developed world, with many implications for health services. The migration of doctors from low and middle income countries to high income countries in North America and Western Europe is well documented [2–4]. Political rhetoric has raised concerns for immigrant doctors in, for example, the United States [5–7]. With developed countries often dependent on healthcare professionals who trained abroad [8], the question of how to assess qualifications from another country is significant. Healthcare services need to ensure that staff are competent and that mechanisms to ensure this are transparent to the public. At present, in the UK, doctors who qualified in the rest of the EU, the wider European Economic Area (EEA) or in former EEA member Switzerland can practise without further assessment of their clinical skills. As shorthand, we refer to these below as EEA doctors. Those trained elsewhere must pass the Professional and Linguistic Assessments Board (PLAB) test before practising: we refer to these non-EEA, international medical graduates as IMGs. See Table 1 for a summary. The regulatory context in the future is as yet unknown [9, 10].

**Table 1:** UK licensing arrangements (while the UK is a member of the European Union)

Shorthand used in this paper	Category and notes	Countries (listed by population size)	Licensing arrangements for doctors working in the UK
UK doctors	UK: current member of EU and EEA; due to leave EU; EEA membership plans unknown	UK	Can practise in the UK if qualified in one of these countries
EEA doctors	Remainder of the European Union; all also in the EEA	Germany, France, Italy, Spain, Poland, Romania, Netherlands, Belgium, Greece, Czech Rep., Portugal, Sweden, Hungary, Austria, Bulgaria, Denmark, Finland, Slovakia, Ireland, Croatia, Lithuania, Slovenia, Latvia, Estonia, Cyprus, Luxembourg, Malta	
	Remainder of the EEA; not in the EU	Norway, Iceland, Liechtenstein	
	Former EEA member	Switzerland	
International medical graduates (IMGs)	Rest of the world	Rest of the world	Required to pass the Professional and Linguistic Assessments Board (PLAB) test

There has been a dramatic change in the pattern of non-UK trained doctors joining the medical register in recent years**.** A higher proportion of graduates from the EEA, particularly Spain and Greece, joined the medical register compared to IMGs [5]. Historically, the proportion of doctors in the UK who were IMGs was always higher than EEA doctors. While EEA doctors only represented a tenth of the total medical population in 2013, their proportional increase is likely to be due to the economic downturn in southern Europe and the expansion of the EEA [6]. Similar trends have been noted elsewhere, including Germany [11] and Ireland [2]. Meanwhile, changes to the UK’s immigration policy in 2010 made it more difficult for IMGs to get an employment visa, perhaps explaining the decrease in the proportion joining the register [12].

The General Medical Council (GMC) are the UK’s medical regulatory body responsible for protecting, promoting and maintaining the health and safety of the public. As part of this role, the GMC can investigate the fitness to practise of doctors working in the UK about which they receive complaints to ensure proper standards in medical practice are upheld. Where the concerns relate to an aspect of the doctor’s performance (such as a basic lack of knowledge, poor clinical judgement, inappropriate prescribing, tendency to use outdated techniques or poor record keeping), they may be required to take a performance assessment. A performance assessment may form one part of a wider investigation into a doctor’s fitness to practise.

The GMC holds data on all doctors who have undergone performance assessment as part of a Fitness to Practise investigation. Doctors under performance assessment usually undergo a peer review and test of competence [13, 14]. The test of competence is used to identify potential gaps in a doctor’s knowledge base and/or their clinical skills. This includes a written knowledge test using Single Best Answer format and an Objective Structured Clinical Examination closely tailored to the doctor’s grade, speciality and clinical work. The marks achieved by the doctor under investigation are compared to the range of marks achieved by a comparison group of doctors who have volunteered to take a similar test in the same specialty [15]. Trained investigators at the GMC use all of the above aspects of a performance assessment to reach a judgement about a doctor’s fitness to practise. These data are collated from the LRMP and portfolios of each doctor that contains their personal information, qualifications and details around their employment history and clinical work.

While the UK relies on foreign trained doctors to fill shortages in the health service [8], their clinical performance has been the subject of concern in recent years, arguably unfairly [16]. Poorer performance of IMGs in postgraduate examinations in the UK has been reported in numerous studies when compared to UK graduates [17, 18]. We also know that IMGs are more likely to be complained about to the GMC and receive more severe disciplinary action than other doctors in Fitness to Practise investigations [19, 20]. However concern has also grown around the performance of European trained doctors [20, 21]. This has been highlighted by high profile cases such as the German-trained doctor who killed a patient during his first locum shift in the UK, and a Bosnian-trained obstetrician who caused the death of a new-born baby [22]. Evidence shows these are not isolated incidents. One study analysed results on part one of the Royal College of Anaesthetists examination and produced a list of countries of primary medical qualification (PMQ) that performed worse than UK graduates. While graduates from Egypt, Iraq and Pakistan performed worse, so did graduates from certain European countries [23]. GMC statistics also report that men who graduated from a European medical school are at greater risk of being complained about to and/or disciplined by the GMC [19]. In GMC Fitness to Practise hearings between 2009 and 2012, higher proportions of EEA doctors were found to be impaired in their performance compared to IMGs and UK graduates [24–27]. Further, an earlier study that analysed erasures and suspensions from the List of Registered Medical Practitioners (LRMP) by country of medical qualification, found that European countries made up half of the top 20 countries that were more likely than the UK to have their graduates be erased or suspended by the GMC [28].

We note that this contrasts with the situation in the US where a recent study found that patients treated by international graduates had lower mortality than patients cared for by US graduates [29] and international graduates perform better in some exams [30].

### Aim and purpose of study

Given the recent and likely forthcoming changes to the UK’s medical register and the increasing concern around the performance of non-UK medical graduates, we compared the demographics of the UK’s medical register with doctors who had undertaken a performance assessment with the GMC to determine whether country of training predicts performance assessments and, if so, whether this might be explained by other factors. We focussed on the prevalence of GMC performance assessments by country of PMQ. Data made available by the GMC allowed us to control for some additional factors and examine performance in more detail than previous studies.

## Methods

This was a retrospective cohort analysis of data routinely collected by the GMC.

We compared two sets of data:

1. a cohort of doctors who had a performance assessment between 1996 and 2013.
2. the medical register (LRMP) between 1996 and 2014.

Performance assessment data was only available up to 2013, but LRMP data was additionally available for 2014. The cohorts were compared according to: sex, country and year of primary medical qualification, specialty and age.

### Variables

The data on sex was complete for both cohorts. In order to plot trends over time, we analysed the year the GMC received an enquiry about each doctor; this date marked the start of their performance investigation. There were 29 cases for which the enquiry year was missing. We also analysed the medical register at each year end.

Data was available for the country and year doctors gained their PMQ from. Data was also recorded on specialty and age band.

We did not have data on the outcome for those under performance investigation. Investigations take some time to complete and many were not complete at the time of data collection. From other GMC data, we know that among individuals who complete tests of competence, over half receive some kind of sanction, with roughly equal numbers being cleared without sanction or voluntarily withdrawing from the register [31].

### Data analysis

We analysed the demographics of the two cohorts by comparing frequency data. We focused our analysis on doctors’ PMQ country, initially in the context of changes to the medical register over time. We then compared the rates of performance assessments over time by PMQ country using negative binomial regression. This allows us to consider country differences while adjusting for the significant changes in the rate of assessments over time.

## Results

### Comparing performance assessment cases with medical register

We compared the population of doctors under performance assessment with doctors who were on the medical register. We show descriptive statistics in order to provide due context for the analysis by country of training. Results for the earliest (1996) and latest (2014) years in the data are shown in Table 1. Men and black minority ethnic doctors were over represented as compared to the medical register. More than 80% of doctors under performance assessment were above 40 years old, as compared to the medical register where a large proportion of doctors are under 40. A significantly higher number of surgeons and general practitioners had a performance assessment as compared to the proportion listed on the medical register as of 2014 (Table 2).

**Table 2:** Comparing demographics of doctors under GMC’s performance assessment with UK medical register between years 1996–2014

		Doctors under performance assessment between 1996 and 2013	Doctors on medical register in 1996	Doctors on medical register in 2014
Sex	M	919 (83%)	125,980 (69%)	148,562 (56%)
	F	192 (17%)	56,570 (31%)	118,597 (44%)
Ethnicity				
White		450 (41%)	72,909 (40%)	139,177 (52%)
Black minority ethnic		661 (59%)	19,419 (11%)	78,285 (29%)
Unknown		0	90,229 (49%)	49,697 (19%)
Age				
21–30		17 (2%)	30,600 (17%)	42,790 (16%)
31–40		109 (10%)	47,422 (26%)	80,839 (30%)
41–50		257 (23%)	33,846 (19%)	67,322 (25%)
51–60		322 (29%)	20,893 (11%)	46,223 (17%)
61–70		208 (19%)	16,121 (9%)	19,733 (7%)
71–80		32 (3%)	8,087 (4%)	4,965 (2%)
81+		n/a	1,557 (<1%)	1,210 (<1%)
Unknown		163 (15%)	24,031 (13%)	4,077 (2%)
Specialty				
Acute medicine		67 (6%)	4,441 (14%)	13,187 (9%)
Medicine		134 (12%)	10,311 (33%)	29,872 (20%)
Surgery		202 (18%)	7,063 (23%)	19,319 (13%)
Psychiatry		49 (4%)	3,581 (12%)	9,365 (6%)
GP		493 (44%)	^a^ n/a	65,127 (44%)
Other		20 (2%)	5,634 (18%)	11,088 (7%)
Unspecified		146 (13%)	0	0
TOTAL		1111	31,030	147,958

^a^ GP register only began in 2006

### Comparing PMQ between performance assessment cases and medical register

Doctors on the medical register in 2014, the most recent year of the data, received their PMQ from 149 different states or territories. Doctors under performance assessment from1996 to 2013 qualified from 58 states or territories. Table 3 shows the 25 countries with the most performance assessments. Doctors on the medical register in 2014 most frequently graduated from the UK, India and Pakistan. The highest number of performance assessments was also conducted on UK, Indian and Pakistan graduates (Table 3).

**Table 3:** Comparing the UK medical register with GMC’s performance assessment cases against the most frequent 25 countries of PMQ

Rank	Country of PMQ	Number of registered Doctors (2014)	Rank	Country of PMQ	Number of performance assessment cases (1996–2013)
1	United Kingdom	169,239	1	United Kingdom	332
2	India	25,001	2	India	242
3	Pakistan	9786	3	Pakistan	61
4	South Africa	5276	4	Nigeria	47
5	Nigeria	4185	5=	Egypt	45
6	Ireland	4042	5=	Germany	45
7	Italy	3539	7	Iraq	30
8	Greece	3423	8	Ireland	28
9	Egypt	3336	9	Bangladesh	23
10	Germany	3241	10	Sri Lanka	21
11	Sri Lanka	2421	11	South Africa	17
12	Iraq	2382	12	Romania	16
13	Romania	2315	13	Poland	15
14	Poland	2091	14=	Italy	14
15	Australia	1955	14=	Spain	14
16	Spain	1745	16	Greece	12
17	Sudan	1650	17	France	11
18	Hungary	1496	18	Myanmar	9
19	Czech Republic	1235	19=	Czech Republic	7
20	Bulgaria	969	19=	Sudan	7
21	Bangladesh	889	21=	Belgium	6
22	Netherlands (excluding Saba)	846	21=	Hungary	6
23	Russia	806	21=	Iran	6
24	Jamaica	800	24=	Austria	5
25	Libya	755	24=	Bulgaria	5
				Ghana	5
				Netherlands	5

### Changes to the medical register over time

It was necessary to place the performance assessment data in the context of changes that have happened to the medical register between 1996 and 2013. The rate of assessments depends on a denominator, the number of doctors trained in different countries, that has been changing rapidly. We summarise these changes in Table 4 and compared them to the proportion of performance assessments that have been conducted on doctors qualifying in the same regions. This gave us a sense of which places of qualification are over and underrepresented in the GMC’s performance assessments when compared to the medical register. Based on the data available, we decided to analyse the data for the following 16 countries and categories:

**Table 4:** Comparing place of doctor’s qualification between UK medical register and GMC’s performance assessment data

Country	Number of registered doctors in 2013 *N* = 259,642	Absolute change on register since 1996	Percentage change on register since 1996	Total performance assessment cases *N* = 1111
Bangladesh	874 (0.3%)	+253	41%↑	23 (21%)
Egypt	3215 (1%)	+1438	81%↑	45 (5%)
Germany	3258 (1%)	+808	33%↑	45 (5%)
Greece	3077 (1%)	+1791	139%↑	12 (1%)
India	25,114 (10%)	+12361	97%↑	242 (22%)
Iraq	2326 (0.8%)	+1417	156%↑	30 (3%)
Ireland	4020 (2%)	−2689	40%↓	28 (3%)
Italy	2917 (1%)	+1973	209%↑	14 (1%)
Nigeria	4067 (2%)	+3049	300%↑	47 (4%)
Pakistan	9400 (4%)	+6686	246%↑	61 (5%)
South Africa	5444 (2%)	−880	14%↓	17 (2%)
Sri Lanka	2376 (0.9%)	+82	4%↑	21 (2%)
United Kingdom	164,691 (63%)	+39205	31%↑	332 (30%)
^a^ EU 2004 states	6206 (2%)	+5513	796%↑	33 (3%)
EEA remainder	7715 (3%)	+4638	151%↑	67 (6%)
Rest of the world	14,942 (6%)	+1532	11%↑	70 (6%)

^a^ Cyprus, Czech Rep., Estonia, Hungary, Latvia, Lithuania, Malta, Poland, Slovakia, Slovenia

I). the UK
II). the ten countries with the highest numbers of registered doctors in 2014
III). the ten countries with highest number of performance assessments (which adds Bangladesh, Iraq and Sri Lanka in addition to the above)
IV). European Union (EU) 2004 accession states as a block, i.e. countries that joined the EU in 2004, namely Cyprus, Czech Republic, Estonia, Hungary, Latvia, Lithuania, Malta, Poland, Slovakia, and Slovenia
V). a combined category for all other current EEA states not otherwise covered plus Switzerland (i.e., countries excluded from the Professional and Linguistics Assessment Board examination)
VI). rest of the world (ROW)

Small numbers prevented us from considering other countries in detail.

The total number of UK trained doctors has increased on the medical register by 31% since 1996 (from 125,486 to 164,691, Table 4). For other countries, the largest absolute increase is Indian-trained doctors, up 12,361(97%), with the largest fall being Australian-trained, down to 2868 (from 4834 in 1996 to 1955 in 2014). The largest number of performance assessments was conducted on UK graduates (332), but the proportion of assessment cases on UK graduates is less than half that of the proportion on the medical register in 2013. UK graduates are underrepresented in performance assessments.

**Fig. 1:**
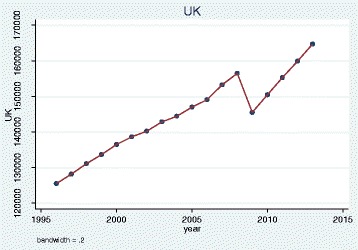
Pattern of UK trained doctors joining medical register between 1996 and 2014

**Fig. 2:**
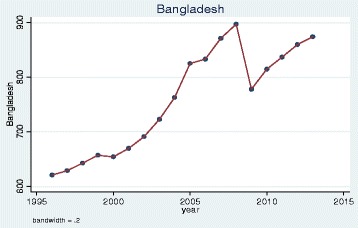
Pattern of Bangladesh trained doctors joining medical register between 1996 and 2014

**Fig. 3:**
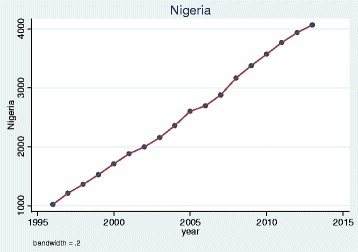
Pattern of Nigerian trained doctors joining medical register between 1996 and 2014

**Fig. 4:**
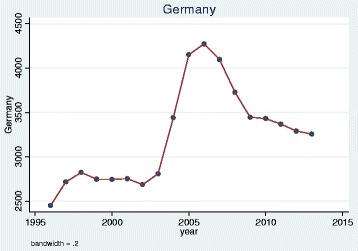
Pattern of German trained doctors joining medical register between 1996 and 2014

**Fig. 5:**
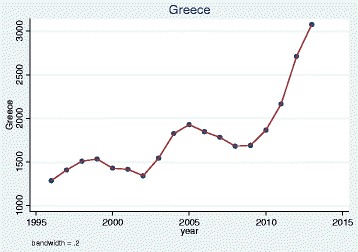
Pattern of Greek trained doctors joining medical register between 1996 and 2014

**Fig. 6:**
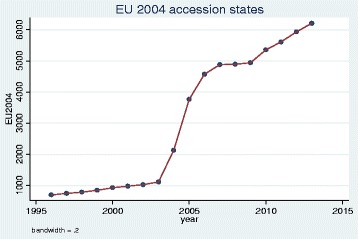
Pattern of doctors who trained in a state that became part of EU in 2004 who joined medical register between 1996 and 2014

We plotted trends over time and, as illustrative examples, show the results for doctors qualified in the UK, Bangladesh, Germany, Greece, Nigeria and the EU 2004 states. The pattern of UK and Bangladesh trained doctors was similar: a steady increase in doctors joining the register until a sharp decrease between 2008 and 2009 (Figs. 1 and 2). Whereas doctors that trained in Germany, Greece, Nigeria and the EU 2004 states differed dramatically from one another in their patterns of joining the medical register (Figs. 3, 4, 5 and 6). In particular, the effects of the economic crisis are evident in the recent sharp increase in Greek trained doctors joining the UK medical register (Fig. 5).

### Comparing rates of performance assessments by PMQ

We calculated the rate of performance assessments per thousand doctors, per enquiry year. Table 5 shows an inverted-U shape across time. The highest rate of performance assessments was conducted on doctors who trained in Bangladesh in 2001 and 2002 (roughly 4.5 cases per thousand per year). Doctors who trained in the UK and South Africa showed consistently low rates of performance assessments across the time period (Table 5).

**Table 5:**
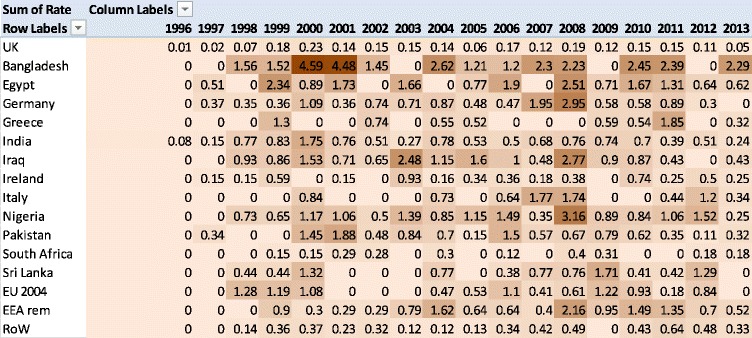
Heat map of rates of performance assessments by place of qualification (darker shades represent higher rates of performance assessments)

After some exploratory analyses, we performed a negative binomial regression on the number of assessments and number of doctors on the register in each year by PMQ country. The data were over-dispersed compared to a Poisson regression, so a negative binomial regression was used. We included independent variables to cover: (1) year (modelled as a quadratic function), and (2) place of medical qualification. This allows us to control for changes over time in assessment rates and the make-up of the medical register. The model was highly significant: *χ* ^2^(17) = 188, *p* < 0.0001, pseudo-R^2^ = 15%. The rate of performance assessment varies significantly by where a doctor qualified and by enquiry year.

**Fig. 7:**
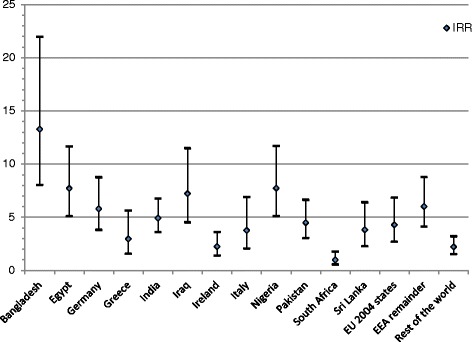
Incidence rate ratios of performance assessments by qualification region with 95% confidence intervals. A ratio of 1 means no difference from the baseline of UK-trained doctors. All regions had statistically significantly different rates of performance assessment with the UK except for South Africa

We calculated an incidence rate ratio (IRR) of performance assessment for PMQ country, with UK graduates as the baseline, controlling for enquiry year (Table 5). An IRR can be interpreted similarly to an odds ratio: 1 represents no difference with UK graduates. All doctors who trained outside the UK were more likely to have a performance assessment, with the exception of South African-trained doctors. Doctors who trained in Bangladesh were 13 times more likely to have a performance assessment than UK graduates, followed by Egyptian and Nigerian trained doctors with an IRR of 8. Doctors who trained in the EU accession states and the remainder of EEA were more than 4 times likelier to have a performance assessment than UK graduates. We plot these incidence rate ratios with confidence intervals in Fig. 7. An important caveat to note is that these IRRs represent very small actual numbers of doctors given the low baseline rate.

We also did pairwise comparisons to test whether the differences in IRRs between PMQ regions were significant or not (Table 6). The rate of performance assessments conducted on UK trained doctors is significantly lower compared to doctors who qualified outside of the UK with the exception of South African-trained doctors (Table 6).

**Table 6:** Pairwise comparisons between each place of medical qualification

Country pairwise comparisons	Bangladesh	Egypt	Germany	Greece	India	Iraq	Ireland	Italy	Nigeria	Pakistan	South Africa	Sri Lanka	EU 2004 states	EEA remainder	Rest of the world
UK	●	●	●	●	●	●	●	●	●	●		●	●	●	●
Bangladesh			●	●	●		●	●		●	●	●	●	●	●
Egypt				●	●		●	●		●	●	●	●		●
Germany							●				●				●
Greece						●			●		●			●	
India							●		●		●				●
Iraq							●				●	●			●
Ireland									●	●	●		●	●	
Italy									●		●				
Nigeria										●	●	●	●		●
Pakistan											●				●
South Africa												●	●	●	●
Sri Lanka															
EU 2004 states															●
EEA remainder															●

● indicates significant difference between the row and column categories at 5% level

### Analysis of the medical register

The GMC provided a list of all doctors who had been on the register at any time between 1996 and 2014 with sex, country of PMQ and year of PMQ. This covers 440,505 individuals. We analysed this data to identify possible confounders to our main analysis.

Using the same country coding as the previous analysis, we investigated whether PMQ region varies by these other variables. There is a significant difference by PMQ region and gender: *χ* ^2^(15) = 7425, *p* < 0.001. Every non-UK region has more male doctors, ranging from 86.4% for Egypt to 58.2% for the EEA remainder category, compared to 56.6% for the UK.

If we compare PMQ country by being on the GP register, there is a significant difference: *χ* ^2^(15) = 22,584, *p* < 0.001. Every non-UK region has fewer doctors on the GP register, ranging from 1.2% for Greece to 18.2% for Bangladesh and Nigeria, compared to 25.6% for the UK.

A Kruskal-Wallis test of PMQ year by PMQ country was statistically significant: *χ* ^2^ (15) = 18,059, *p* < 0.001. This means that on average, the year that doctors qualified varied by where they graduated from. For example, Irish graduates on average (median) qualified the earliest, in 1981, whereas UK graduates on average qualified in 1992. Italian trained doctors qualified the latest, 1999 on average.

## Discussion

### Summary of main results

The rate of performance assessment varies significantly by enquiry year and where a doctor qualified. Controlling for year, doctors who trained outside the UK were more likely to have a performance assessment, with the exception of South African trained doctors. Doctors who trained in Bangladesh were 13 times more likely to have a performance assessment than UK graduates, followed by Nigerian and Egyptian trained doctors who were 8 times more likely. German trained doctors were over represented among EEA graduates. Doctors who trained in the 2004 EU accession states and the bulk of the EEA were over 4 times more likely to have a performance assessment than UK graduates.

### Findings in relation to other studies

Our findings indicate that the prevalence of GMC performance assessments differs depending on a doctor’s country of qualification. Further, our data supports that of other studies that highlight doctors who trained in certain countries within and outside of the EEA perform worse than UK graduates. South African trained doctors, like UK graduates, have been found to perform better than average on part one of the Royal College of Anaesthetists’ examination, while graduates from Greece, Germany and Ireland performed significantly worse [23], matching our results on performance assessments from these countries. Our results also concord with an earlier analysis of the LRMP [28]. While previous evidence has focused on comparing how UK graduates and IMGs compare in the Fitness to Practise process [20], this is the first study since Wakeford’s in 2011 [28] that has explored the process on a country by country basis and the first that has been able to control for changes over time.

Perhaps one explanation is in differences in the way doctors are trained in different countries. Training in the UK has long been integrating patient contact and practical skills into early undergraduate training [32]. German medical schools have only adopted this approach more recently [33]. Questionnaire studies have also reported major differences in medical regulation among non-UK countries. One study found that in certain countries (Spain, Austria, Finland and Estonia) there was an implicit expectation that doctors maintain competence without needing to comply with formal standards. Whereas in others (Belgium, Germany, Hungary, the Netherlands, Slovenia and the UK), an explicit demonstration of continued competence is mandatory [34]. Further, when representatives from 14 EU countries were surveyed, authors concluded that the systems of licensing and registration vary so much that it creates confusion and problems in the context of the free movement of doctors. In particular, there were large differences in how fitness to practise is conceptualised and some countries showed weaknesses in their systems that should identify doctors who are unfit to practise [35]. As well as IMGs, European graduates are more likely to experience difficulty in adapting to a very different healthcare context than the one in which they trained [21]. Another unanswered question is how representative doctors who choose to move to the UK are compared to doctors who stay practicing in the country in which they trained.

There is also a question around who gets complained about to the GMC [16]. Non-UK trained graduates, including those from the EEA as well as minority ethnic doctors, are more vulnerable to being complained about than white UK graduates [36]. It is possible that an over representation of non-UK trained doctors is due to prejudice. Unconscious bias against foreign-born doctors has been shown experimentally among students [37] and staff from ethnic minority backgrounds working in the NHS are more likely to report harassment and discrimination from their patients and colleagues [38]. Similar issues with discrimination by patients and colleagues were seen in a US survey of Muslim doctors [5].

### Meaning of the study

Rates of performance assessments differ depending on the country or region a doctor trained in. However, without further investigation, it is unclear as to whether this represents a true difference in doctors’ competence. Rates of performance assessments overall are very low and absolute differences between countries are tiny. Problems leading to an investigation may not reflect poor medical competence, but be due to poorer English language skills or lack of knowledge of the UK’s health service. Performance investigation is not a reflection of poor performance alone but also of complaints patterns, which could indicate prejudice in the system.

Our findings suggest concern not only with IMGs but also European-trained doctors given that they were significantly more likely to have a GMC performance assessment than doctors trained in the UK, South Africa and our “rest of the world” category. One qualitative study found that European trained doctors reported similar difficulties to IMGs when adapting to practising medicine in the UK’s healthcare setting and medical regulatory system [39]. European law has prevented the selective testing of doctors trained in an EEA country that is different to how UK trained doctors are tested. There is currently no mandatory assessment process to check the competence of EEA medical graduates before their UK medical licence is granted, although we note that EEA graduates who have not done the PLAB broadly did no worse than IMGs who have done the PLAB. The GMC is going to introduce a medical licensing assessment that will be mandatory for UK graduates and IMGs prior to their registration. It was unlikely that the elements of this planned assessment could have been enforced on European graduates given European laws around free movement [19, 40]. Currently, the GMC can only seek evidence of sufficient English language skills in European graduates and can refuse to grant a licence to practise where this is not provided [41]. All this may change in a post-Brexit regulatory environment that is yet to be determined. Further analysis of this topic is needed rapidly to inform decision making.

We also note that not all overseas trained doctors were more likely to have a performance assessment. South African trained doctors were no more likely to have a performance assessment than UK trained doctors. While there were too few Hong Kong trained doctors to include separately in the main analysis—their numbers have decreased from 2730 to 614 between 1996 and 2013—we note none had a performance assessment. Therefore it is a matter of further investigation as to why doctors from certain countries or regions might be more at risk than others of being investigated by the GMC due to performance concerns.

### Strengths and weaknesses of the study

This is the first study to explore whether place of medical qualification on a country by country basis affects the likelihood of having a GMC performance assessment. We urge caution in the interpretation of our findings. It is not true that all doctors who qualified outside the UK are more likely to have a GMC performance assessment and those doctors who have an investigation for performance concerns represent a tiny minority of overseas trained doctors. While the incidence rate ratios appear large for some PMQ countries, the number of doctors investigated for each PMQ country is low and absolute differences between countries are tiny. The results are further complicated because a performance investigation is not a reflection of poor performance alone but also of who gets complained about and how severe the complaint is. A proportion of doctors under performance investigation are found to be fit to practise. It is also likely that some doctors may show performance concerns but that they are being managed locally and do not meet the GMC’s threshold for an investigation. To unpack these factors requires data that we did not have on referral patterns by PMQ country or final outcome of the investigation.

We know that doctors’ sex [42] and age [43] affect performance and those factors vary by where a doctor trained in our analysis of the register data. We also know that complaints vary by specialty, which also varies by where a doctor trained. We have not been able to adjust for these confounders. Therefore differences between PMQ countries may be explainable in terms of the doctors who trained in a particular country having a different profile in terms of sex, age and specialty.

## Conclusions

From 1996 to 2013, doctors that trained outside of the UK, including EEA countries, had significantly higher rates of GMC performance assessments than UK-trained doctors. The reasons for this are unclear without further investigation. Poor performance can manifest itself in many forms some of which include poor clinical knowledge, English language skills and a misunderstanding of patient’s culture or the UK’s healthcare settings. Factors such as sex, age and specialty of doctors are likely to be confounding variables. If differences persisted after controlling for these factors, it could reflect true differences in competency, standards of medical training and/or certification between different countries, or it could reflect different treatment of this group by society and employers [16]. Whether differences by country of training reflect differences in the ability of some doctors, or biases in systems of performance evaluation, this is an important phenomenon that further research needs to explain.

There may be implications for transnational agreements on freedom of movement of healthcare professionals, and for what testing is required by national governments of individuals trained elsewhere. With the UK having to negotiate new arrangements after exiting the EU, such questions have become more urgent. Research in the UK can also inform the situation in other countries facing similar political, economic and social pressures. In the meantime, patients should be reassured that the vast majority of doctors working in the UK, irrespective of where they trained, are competent, and indeed highly skilled. Further, the NHS could not function without foreign-trained doctors or other healthcare professionals.

## Abbreviations

EEA: European Economic Area
EU: European Union
GMC: General Medical Council
IMG: International Medical Graduate
IRR: Incidence rate ratio
LRMP: List of Registered Medical Practitioners
NHS: National Health Service
PLAB: Professional and Linguistics Assessments Board
PMQ: Primary medical qualification
ROW: Rest of the world
UK: United Kingdom
